# The peripartum viral load cascade and outcomes of infants exposed to HIV in Lesotho

**DOI:** 10.1111/hiv.70082

**Published:** 2025-07-26

**Authors:** Kathrin Haenggi, Moleboheng Mokebe, Lipontso Motaboli, Makobefo G. Chakela, Mpho Kao, Mathebe Kopo, Irene Ayakaka, Morongoe Nyakane, Tapiwa Tarumbiswa, Niklaus D. Labhardt, Jennifer A. Brown, Nadine Tschumi

**Affiliations:** ^1^ Division of Clinical Epidemiology, Department of Clinical Research University Hospital Basel Basel Switzerland; ^2^ University of Basel Basel Switzerland; ^3^ SolidarMed, Partnerships for Health Maseru Lesotho; ^4^ Ministry of Health Lesotho Maseru Lesotho

**Keywords:** Africa, antiretroviral therapy, HIV viral load monitoring, postpartum, pregnancy

## Abstract

**Objectives:**

This study describes the peripartum viral load (VL) monitoring cascade among individuals initiating antiretroviral therapy (ART) before delivery, as well as HIV testing, prophylaxis and outcomes of their infants in routine care in Lesotho, southern Africa.

**Methods:**

Data from medical charts review of paper‐based registers at 20 healthcare facilities in two districts of Lesotho were combined with data from the Viral Load Cohort North‐East Lesotho (VICONEL). We included people with HIV who attended their first antenatal care visit at a participating healthcare facility after 31 December 2019, had a delivery date before 1 January 2022 and initiated dolutegravir‐based ART before delivery, as well as their infants.

**Results:**

A total of 353 pregnant people with HIV and 357 infants exposed to HIV were recorded. Among 17/353 (5%) with an initial VL ≥1000 copies/mL, a timely (within 4 months) follow‐up VL was provided for 4/17 (24%). Among 233/353 (66%) with an initial VL <1000 copies/mL, a timely (within 7 months) follow‐up VL was provided for 120/233 (52%). For 103/353 (29%), no VL measurement was available during pregnancy. Overall, peripartum VL monitoring was guideline‐adherent for 157/353 (44%) pregnancies. Among infants exposed to HIV, 301/357 (84%), 274/357 (77%) and 172/357 (48%) received guideline‐adherent prophylactic ART, early infant HIV testing within 2 months and 9‐month HIV testing, respectively. Vertical transmissions occurred in 2/357 (1%) infants.

**Conclusion:**

There were substantial gaps in peripartum HIV service delivery or uptake as well as in HIV prevention and testing of infants exposed to HIV.

## INTRODUCTION

Programmes aiming to prevent vertical transmission of human immunodeficiency virus (HIV) have averted HIV transmission to an estimated 3.4 million children since 2000 [1]. However, antiretroviral therapy (ART) coverage among pregnant people has changed little over the last decade, and the decline in new HIV acquisition in children has slowed considerably. In 2023, there were 120 000 new cases of HIV globally among children below 5 years of age [2].

Vertical transmission of HIV can occur prenatally, during labour or while breastfeeding [3]. A peripartum viral load (VL) ≥1000 copies/mL is the main risk factor for vertical transmission of HIV, and successful ART leading to viral suppression is thus key for prevention [4, 5]. ART reduces vertical transmission from about 20%–45% to below 1% if viral suppression is achieved, though overall vertical transmission rates remain between 1% and 10% in Southern African countries [6, 7]. Breastfeeding is critical for child health in resource‐limited settings [8] and the World Health Organization (WHO) recommends breastfeeding beyond 12 months for people who are taking ART [4, 8, 9]. Hence, maintaining viral suppression throughout pregnancy and breastfeeding is key to eliminating vertical transmission [10].

The WHO lists the VL monitoring algorithm in pregnant and breastfeeding people receiving ART as a key research gap [4]. There are limited data from routine care in resource‐limited settings on adherence to VL monitoring algorithms and on overall service delivery within this subpopulation. This study aims to describe adherence to guideline‐recommended VL monitoring and the infant HIV testing schedule in routine care, focusing on pregnancies where ART was initiated prior to delivery in two districts of Lesotho, southern Africa.

## METHODS

### Study design

This study combined a retrospective medical charts review with data from the Viral Load Cohort North‐East Lesotho (VICONEL) to describe the peripartum VL monitoring cascade as well as HIV testing, prophylaxis and outcomes of infants exposed to HIV among people in care in Butha‐Buthe and Mokhotlong districts, Lesotho. Specifically, it evaluated whether service delivery was guideline‐adherent. Reporting follows the STROBE statement [11].

### Setting

Lesotho has the second‐highest adult HIV prevalence worldwide at 19% in 2023, with women being disproportionately affected [12, 13]. Approximately 23% of pregnant people attending antenatal care (ANC) are living with HIV [14], and the rate of vertical transmission is estimated at 5% [12]. ART provision is mostly nurse‐led. According to national guidelines, peripartum people with HIV should undergo more intensive VL monitoring compared to the general population [14, 15, 16]. During the study period, the guidelines recommended VL testing every 6 months during the peripartum period (as opposed to every 12 months for adults who were not pregnant or breastfeeding) if the VL was <1000 copies/mL, with a VL ≥1000 copies/mL triggering enhanced adherence counselling and follow‐up VL testing within 8–12 weeks (as for the overall population). For people with sustained viraemia ≥1000 copies/mL despite adequate adherence, national guidelines recommend HIV resistance testing to inform onward care. During the study period, new national guidelines recommended classifying infants exposed to HIV as at high risk if ART was only initiated in the third trimester, or if the last VL in the third trimester was either ≥1000 copies/mL or missing [17]. HIV testing of infants exposed to HIV is recommended within 6 weeks and at 9 months after birth as well as at 18 months after birth or 3 months after cessation of breastfeeding, with additional testing for infants at high risk [15, 16, 17].

### Participants

This study includes people with HIV who were pregnant and attended their first antenatal care visit at one of 20 participating healthcare facilities after 31 December 2019, had a delivery date before 1 January 2022 and initiated dolutegravir‐based ART before delivery. These criteria aim to reflect the reality in the era of dolutegravir‐based ART while allowing sufficient time for outcome ascertainment. Furthermore, this study includes infants of eligible people. Pregnant people were excluded if their data could not be matched to infant data and vice versa. Parent‐infant pairs were also excluded in the case of known infant death within 24 months, as this altered postpartum VL monitoring algorithms for the parent and infant HIV testing and outcomes could no longer be ascertained. Pregnant and breastfeeding people were followed up from their first ANC visit until 6 months postpartum, whereas the follow‐up period for infants exposed to HIV was 24 months after birth.

### Data sources/measurements

VL and treatment‐related data were derived from the VICONEL database, which has been described previously [18].

VICONEL data were complemented by retrospectively collected data from paper‐based entries in the ANC, delivery, postnatal care, under‐5 and HIV‐exposed infant registers available at all healthcare facilities. All paper‐based data from registers were collected and entered into a dedicated REDCap electronic database [19, 20].

### Variables/statistical methods

Pregnancy start and related variables were defined using the best available estimate from the following indicators with decreasing accuracy: expected date of delivery by ultrasound scan, last normal menstrual period (LNMP), expected date of delivery by LNMP, delivery date or gestational age at first ANC visit. The first, second and third trimesters were defined as LNMP until <14 weeks, ≥14 to <27 weeks and ≥27 weeks to date of delivery, respectively.

Among peripartum people with HIV, we considered a follow‐up VL test 3 months (window: 0–4 months) after an initial VL ≥1000 copies/mL or after ART initiation during pregnancy and 6 months (window: 0–7 months) after an initial VL <1000 copies/mL during pregnancy as guideline‐adherent. Among infants exposed to HIV, a first early infant HIV test within a window of 2 months after birth (guideline specifications: testing within 6 weeks) and a second HIV test at 9 months (window: 8–10 months) were considered guideline‐adherent [15, 16]. Prophylaxis in infants exposed to HIV was considered timely if nevirapine prophylaxis was initiated within 2 days (guideline specifications: at birth [16]) followed by cotrimoxazole prophylaxis initiation within 2 months (guideline specification: within 6 weeks [16]). For the 24‐month HIV outcome, we considered the first positive infant HIV test or the last negative test taken beyond 9 months (window: 8–24 months), respectively. A month was defined as 30 days.

All variables were summarized as frequencies and percentages for categorical variables and as medians and interquartile ranges (IQR) for continuous variables. All analyses were done using STATA version 16.0.

### Ethical considerations

This study, as well as the VICONEL cohort, was approved by the National Health Research Ethics Committee in Lesotho (ID212‐2023, approved 29.11.2023 and ID134‐2016, last renewal 20.11.2024, version ID134‐2016‐Modify 02). A waiver of consent was obtained for all data included in the present study.

## RESULTS

### Study population

**FIGURE 1:**
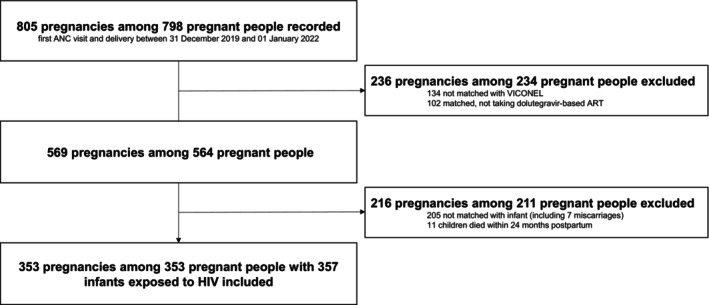
Study flow chart. ANC, antenatal care; ART, antiretroviral therapy; HIV, human immunodeficiency virus; VICONEL, viral load cohort north‐east Lesotho.

A total of 805 pregnancies of 798 people with HIV with a first ANC care visit and delivery between 31 December 2019 and 1 January 2022 were recorded. Of these, 353 (44%) pregnancies among 353 pregnant people with HIV and 357 infants exposed to HIV fulfilled the inclusion criteria. Notably, the excluded pregnancies included 7 miscarriages and 11 additional pregnancies with infant deaths (Figure 1). Among these 11, 4 tested negative for HIV, and for 7 (5 with death at delivery, 2 with unknown date of death), no data on HIV testing were available.

### Characteristics of peripartum parents

Among the 353 pregnant people, the first ANC visit was at a median gestational age of 21 (IQR: 14–27) weeks, and a median of 3 (IQR: 2–4) ANC visits were attended. The median age at delivery was 30 years (IQR: 25–35) (Table 1). ART was initiated before or during 263/353 (75%) and 90/353 (25%) pregnancies, respectively. The median time on ART at delivery was 3 (IQR: 1–6) years. In nearly all cases (352/353, 100%), ART at delivery consisted of tenofovir disoproxil fumarate/lamivudine/dolutegravir. No parental deaths were recorded during delivery and through 24 hours postpartum.

**TABLE 1:** Characteristics of pregnant people with HIV and viral load monitoring during pregnancy and postpartum.

	*N* = 353
Characteristics of peripartum parent	
Age at delivery, *n* (%)	
15–19 years	26 (7%)
20–29 years	144 (41%)
30–39 years	155 (44%)
≥40 years	28 (8%)
Parity at first ANC visit, *n* (%)	
0	64 (18%)
1	110 (31%)
2	87 (25%)
3	44 (12%)
≥4	48 (14%)
Gestational age at first ANC visit in weeks, median (IQR)	21 (14–27)
WHO clinical staging at first ANC visit, *n* (%)	
1	340 (96%)
Missing/not documented	13 (4%)
Number of ANC visits attended, median (IQR) [range]	3 (2–4) [1–7]
HIV and ART history	
History of viremia before pregnancy, *n* (%)	
Always VL <50 copies/mL (no history of viraemia)	138 (39%)
Always VL <1000, at least one VL ≥50 copies/mL	42 (12%)
At least one VL ≥1000 copies/mL	22 (6%)
No VL before pregnancy/not documented	151 (43%)
ART initiated, *n* (%)	
Before pregnancy	263 (75%)
During pregnancy	90 (25%)
Years taking ART at delivery, median (IQR)	3 (1–6)
ART regimen at delivery, *n* (%)	
TDF‐3TC‐DTG	352 (100%)
ABC‐3TC‐DTG	1 (0%)
Viral load testing and outcomes	
Number of VL tests, median (IQR) [range]	
During pregnancy	1 (0–1) [0–3]
0–6 months postpartum	1 (0–1) [0–2]
Throughout pregnancy and 6 months postpartum	1 (1–2) [0–4]
Timing of first VL after pregnancy start, *n* (%)	
First trimester	80 (23%)
Second trimester	89 (25%)
Third trimester	81 (23%)
≤6 months postpartum	78 (22%)
No VL ≤6 months postpartum/not documented	25 (7%)
Timing of first VL if ART initiated during pregnancy, *n* (%)	*n* = 90
In time (≤7 months after ART initiation)	66 (73%)
Late/no VL/not documented	24 (27%)
VL in copies/mL during pregnancy and through 6 months postpartum, *n* (%)	
Always VL <50 copies/mL	267 (76%)
Always VL <1000, at least one VL ≥50 copies/mL	39 (11%)
At least one VL ≥1000 copies/mL	21 (6%)
No documented VL during pregnancy or ≤6 months postpartum	26 (7%)
VL in copies/mL at first VL during pregnancy, *n* (%)	
<50 copies/mL	208 (59%)
50–999 copies/mL	25 (7%)
≥1000 copies/mL	17 (5%)
No documented VL during pregnancy	103 (29%)
Follow‐up VL if VL <1000 copies/mL at first VL, *n* (%)	*n* = 233
In time (≤7 months)	120 (52%)
Late/not done/not documented	113 (48%)
Follow‐up VL if VL ≥1000 copies/mL at first VL, *n* (%)	*n* = 17
In time (≤4 months), resuppressed to <1000 copies/mL	2 (12%)
In time (≤4 months), sustained VL ≥1000 copies/mL	2 (12%)
Late/not done/not documented	13 (76%)

Abbreviations: 3TC, lamivudine; ABC, abacavir; ANC, antenatal care; ART, antiretroviral therapy; DTG, dolutegravir; IQR, interquartile range; NVP, nevirapine; TDF, tenofovir disoproxil fumarate; VL, viral load; WHO, World Health Organization.

### Peripartum VL monitoring

**FIGURE 2:**
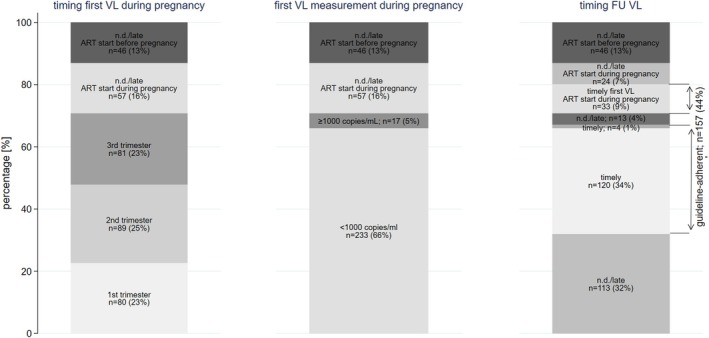
Viral load monitoring cascade and follow‐up outcome of the peripartum parent. ART, antiretroviral therapy; FU, follow‐up; n.d., not done; VL, viral load.

The first VL test during pregnancy was conducted in the first, second and third trimester in 80/353 (23%), 89/353 (25%) and 81/353 (23%) pregnancies, respectively (Figure 2). The proportion without a VL result during pregnancy was 103/353 (29%) overall, 46/263 (17%) among those with ART initiated before pregnancy, and 57/90 (63%) among those with ART initiated during pregnancy. Among 233/353 (66%) pregnancies with a first VL <1000 copies/mL, a timely (window: 0–7 months) follow‐up VL was provided for 120/233 (52%). Among 17/353 (5%) pregnancies with a first VL ≥1000 copies/mL, a timely (window: 0–4 months) follow‐up VL was provided for 4/17 (24%). Two people experienced sustained viraemia ≥1000 copies/mL at the follow‐up measurement. Among those with ART initiation during pregnancy, 66/90 (73%) received a timely (window: 0–7 months) first VL measurement (33 during pregnancy, 33 postpartum). Overall, peripartum VL monitoring was guideline‐adherent for 157/353 (44%) pregnancies (Figure 2).

### Characteristics, HIV testing and outcomes of infants exposed to HIV

**FIGURE 3:**
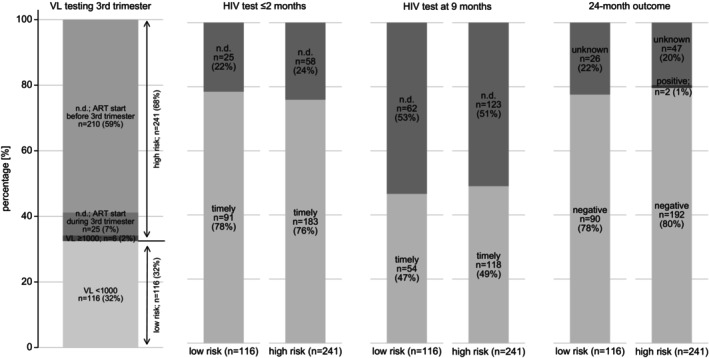
HIV testing and outcomes of infants exposed to HIV. ART, antiretroviral therapy; n.d., not done; VL, viral load [copies/mL].

A total of 357 infants exposed to HIV (349 singletons and 4 sets of twins) were included. Among them, 241/357 (68%) were classified as at high risk for vertical transmission. Table 2 displays their characteristics as well as their HIV testing, prophylaxis and outcomes stratified by risk category. A timely (window: 0–2 months) early infant HIV test was provided for 274/357 (77%). At 9 months (window: 8–10 months), 172/357 (48%) received an HIV test. Nevirapine prophylaxis and cotrimoxazole prophylaxis were initiated in time for 301/357 (84%; window: 0–2 days) and 282/357 (79%; window: 0–2 months), respectively. Two of the 357 (1%) infants tested positive for HIV within 24 months; both were classified as at high risk (Figure 3). These vertical transmissions occurred after ART initiation in the 21st and 34th gestational week, respectively. In both cases, no VL was available during pregnancy, and both infants received nevirapine prophylaxis within 2 days. The first infant tested positive for HIV at 6 weeks while being exclusively breastfed. The first parental VL was ≥1000 copies/mL at 19 weeks postpartum. The second infant tested positive for HIV at 18 months after two negative HIV tests at 6 weeks and at 9 months. This infant was exclusively breastfed at 6 weeks, and the feeding status was unknown at 6 months. There were 2 parental VLs below the detectable limit at 7 and 20 months postpartum.

**TABLE 2:** Characteristics, HIV testing, prophylaxis and outcomes of infants exposed to HIV.

	High risk, *N* = 241	Low risk, *N* = 116	Total, *N* = 357
Characteristics of infants exposed to HIV			
Risk criteria (multiple may apply)			
VL <1000 copies/ml in 3rd trimester, ART initiation before 3rd trimester	0 (0%)	116 (100%)	116 (32%)
No VL in 3rd trimester	232 (96%)	0 (0%)	232 (65%)
VL ≥1000 copies/ml in 3rd trimester	6 (2%)	0 (0%)	6 (2%)
ART initiation in 3rd trimester	28 (12%)	0 (0%)	28 (8%)
Sex, *N* (%)			
Female	118 (49%)	62 (53%)	180 (50%)
Male	123 (51%)	54 (47%)	177 (50%)
Number of infants, *N* (%)			
Singleton	233 (97%)	116 (100%)	349 (98%)
Twin	8 (3%)	0 (0%)	8 (2%)
Gestational age at delivery^a^, *n* (%)			
Pre‐term	51 (21%)	17 (15%)	68 (19%)
Term	182 (76%)	92 (79%)	274 (77%)
Post‐term	8 (3%)	7 (6%)	15 (4%)
Breastfeeding at 6 weeks, *n* (%)			
Exclusive breastfeeding	191 (79%)	89 (77%)	280 (78%)
Exclusive replacement feeding	5 (2%)	1 (1%)	6 (2%)
Mixed feeding	0 (0%)	0 (0%)	0 (0%)
Unknown/not documented	45 (19%)	26 (22%)	71 (20%)
Breastfeeding at 6 months, *n* (%)			
Exclusive breastfeeding	28 (12%)	17 (15%)	45 (13%)
Exclusive replacement feeding	16 (7%)	5 (4%)	21 (6%)
Mixed feeding	77 (32%)	34 (29%)	111 (31%)
Unknown/not documented	120 (50%)	60 (52%)	180 (50%)
HIV testing, prophylaxis and outcome			
Timing of NVP infant prophylaxis, *n* (%)			
In time (≤2 days)	201 (83%)	100 (86%)	301 (84%)
Late (>2 days)	21 (9%)	11 (9%)	32 (9%)
Not received/not documented	19 (8%)	5 (4%)	24 (7%)
Timing of CTX infant prophylaxis, *n* (%)			
In time (≤2 months)	187 (78%)	95 (77%)	282 (79%)
Late (>2 months)	24 (10%)	11 (9%)	35 (10%)
Not received/not documented	30 (12%)	10 (7%)	4′ (11%)
Median number of HIV tests received <24 months, median (IQR) [range]	3 (2–3) [1–5]	3 (2–3) [0–4]	3 (2–3) [0–5]
Early infant HIV test received, *n* (%)			
In time (≤2 months)	183 (76%)	91 (78%)	274 (77%)
Delayed (2–8 months)	49 (20%)	23 (20%)	72 (20%)
Not received/not documented	9 (4%)	2 (2%)	11 (3%)
HIV test at 9 months received, *n* (%)			
In time (8–10 months)	118 (49%)	54 (47%)	172 (48%)
Delayed (10–24 months)	76 (32%)	36 (31%)	112 (31%)
Not received/not documented	47 (20%)	26 (22%)	73 (20%)
HIV status at 24 months, *n* (%)			
Positive	2 (1%)	0 (0%)	2 (1%)
Negative^b^	192 (80%)	90 (78%)	282 (79%)
Unknown/not documented	47 (20%)	26 (22%)	73 (20%)

Abbreviations: ART, antiretroviral therapy; CTX, cotrimoxazole; IQR, interquartile range; NVP, nevirapine; VL, viral load.

^a^ For one infant, the date of delivery was estimated based on LMNP.

^b^ Median age at last HIV test: 19 months (interquartile range: 18–20).

## DISCUSSION

This study assessed VL monitoring during pregnancy and postpartum, as well as HIV testing, prophylaxis and outcomes of their infants exposed to HIV in the context of ART initiation before delivery, among people in care in two districts of Lesotho. It identified substantial gaps in service delivery or uptake. Among 353 pregnancies, guideline‐adherent VL monitoring during pregnancy and postpartum was only provided in 44% of pregnancies. Even among the 75% of pregnancies with ART initiation prior to pregnancy, 17% had no VL testing during pregnancy. Furthermore, 11% of people with HIV had at least one VL 50–999 copies/mL and 6% at least one VL ≥1000 copies/mL during pregnancy and postpartum. Among infants exposed to HIV, 77% and 48% received guideline‐adherent HIV testing before 2 months and at 9 months, respectively. Two infants—both of whom were classified as at high risk for vertical transmission according to national guidelines—were diagnosed with HIV.

Previous studies in this setting have described similar gaps in VL monitoring and clinical action upon detection of viraemia in paediatric and adult care [21, 22]. Insufficient VL testing in pregnant people has also been described in other African settings [23, 24, 25, 26]. The national guidelines of Lesotho have been amended since the study period to lower the VL threshold for clinical action for all people with HIV, while increasing the frequency of VL testing among pregnant and breastfeeding people. For pregnant and breastfeeding people, VL measurements should now be repeated every 3 months if the VL is <50 copies/mL or within 1 month after a VL ≥50 copies/mL [14]. This is slightly more frequent than recommended by the current WHO guidelines, which list the ideal peripartum monitoring algorithm as a key research gap [4, 27]. A recent randomized trial observed faster detection of viraemia, but no difference in subsequent viral suppression between 3‐monthly point‐of‐care versus 6‐monthly laboratory‐based postpartum VL testing [28]. While guideline adherence in the present study was suboptimal already with less‐frequent VL monitoring requirements, it remains to be seen whether increasing the recommended frequency of monitoring might improve the overall VL coverage. Factors such as supply chain challenges and stockouts, which are already common in settings like Lesotho, will need to be addressed to ensure the successful implementation of the new guidelines. The low proportion of guideline‐adherent HIV tests among infants exposed to HIV in our study aligns with observations in other countries in southern Africa in 2020 [29] and indicates that vertical transmissions might have been missed. The observed vertical transmission rate of 1% among pregnant people in care and taking ART at delivery is comparable to previously reported rates with ART [30, 31, 32, 33]. Importantly, the overall rate of vertical transmission in Lesotho regardless of pre‐delivery ART is around 5% according to the most recent UNAIDS data [7]. Assessing gaps in testing and ART coverage among peripartum people was beyond the scope of our study, though the lower rate observed in our study (including only pregnancies with ART initiation before delivery) suggests these factors contribute substantially to overall vertical transmission. Both peripartum VL monitoring and infant HIV testing could potentially be enhanced through broader implementation of point‐of‐care testing, which could thus play a crucial role towards the goal of eliminating vertical transmission of HIV [4, 27].

This study has several limitations. First, it only includes pregnant people who had at least one documented VL measurement since 2015 (VICONEL inclusion criterion), attended ANC care and initiated ART prior to delivery. Hence, our findings relate to pregnant people in HIV care as well as their infants and cannot be extrapolated to the broader population, notably to pregnant people with undiagnosed or untreated HIV. Second, retrospective studies bring the potential for information bias due to incomplete documentation and missing data. Third, additional data gaps emerged from incomplete matching between the registers due to missing or incorrectly captured information. We were unable to compare VL monitoring data between included and excluded individuals, as VL data were obtained from VICONEL and were thus unavailable for unmatched individuals. Fourth, breastfeeding status could often not be verified beyond 6 months. Therefore, we only assessed VL monitoring for the first 6 months postpartum, and the impact of breastfeeding on vertical transmission could not be analysed in detail. Fifth, COVID‐19‐related restrictions in Lesotho during the study period may have impacted healthcare access and guideline adherence.

In conclusion, our findings reiterate previously reported gaps in the implementation of VL monitoring even in the priority population of pregnant and postpartum people with HIV. In combination with incomplete HIV testing among infants exposed to HIV, these gaps reflect a missed opportunity to improve the prevention of vertical transmission of HIV. Evidence is needed to inform the optimal treatment monitoring algorithms and thereafter, efforts to ensure their implementation.

## AUTHOR CONTRIBUTIONS

K.H., J. A. B. and N. T. conceptualized the study. K.H., M.M, M.G.C., M.K., M.M and L.M. trained the study staff and oversaw data collection. M.G.C. and L.M. are responsible for data management in VICONEL. K.H. conducted the data analyses. I.A. is the local principal investigator and N.D.L the sponsor‐investigator of VICONEL. M.N. and T.T. assured alignment of the study with the national HIV programme. K.H., J. A. B. and N. T. wrote the first manuscript draft. All authors approved the final version of the manuscript.

## FUNDING INFORMATION

This study was funded by the Swiss National Science Foundation (grant numbers PCEFP3_181355 and IZ07Z0_160876/1, to NDL) and the Moritz Straus‐Foundation. JAB was supported by the University of Basel Research Fund Junior Researchers (3ZX1422, to JAB) and the Swiss National Science Foundation (P500PM_221966, to JAB) and received part of her salary through a grant from Fondation Botnar (REG‐19‐008, to NDL and JAB).

## CONFLICT OF INTEREST STATEMENT

N.D.L reports having received travel grants to attend scientific conferences from Gilead Sciences and ViiV Healthcare. In 2022 and 2023, his division at the University Hospital Basel received honoraria from ViiV Healthcare. All other authors declare no conflicts of interest.

## ETHICS STATEMENT

The VICONEL cohort (ID134‐2016, last renewal 16.05.2023), from which viral load and treatment‐related data were derived, as well as this study (ID212‐2023, dated 29.11.2023) have been approved by the National Health Research Ethics Committee in Lesotho.

## PATIENT CONSENT STATEMENT

Individual informed consent was waived for the reporting of routine data within this study.

## Data Availability

The data that support the findings of this study are available from the corresponding author upon reasonable request.
